# Cardiotoxicity of Selected Vascular Endothelial Growth Factor Receptor Tyrosine Kinase Inhibitors in Patients with Renal Cell Carcinoma

**DOI:** 10.3390/biomedicines11010181

**Published:** 2023-01-11

**Authors:** Beata Franczyk, Jacek Rysz, Janusz Ławiński, Aleksandra Ciałkowska-Rysz, Anna Gluba-Brzózka

**Affiliations:** 1 Department of Nephrology, Hypertension and Family Medicine, Medical University of Lodz, 113 Żeromskiego Street, 90-549 Lodz, Poland; 2 Department of Urology, Institute of Medical Sciences, Medical College of Rzeszow University, 35-055 Rzeszow, Poland; 3 Palliative Medicine Unit, Oncology Department, Medical University of Lodz, 90-419 Lodz, Poland

**Keywords:** cardiotoxicity, RCC, metastasis, VEGFR-TKIs

## Abstract

Renal cell carcinoma (RCC) is one of the most frequent malignant neoplasms of the kidney. The therapeutic options available for the treatment of advanced or metastatic RCC include vascular endothelial growth factor receptor (VEGFR)-targeted molecules, for example, tyrosine kinase inhibitors (TKI). Various VEGFR-TKIs proved to be effective in the treatment of patients with solid tumours. The combination of two drugs may prove most beneficial in the treatment of metastatic RCC; however, it also enhances the risk of toxicity compared to monotherapy. Specific VEGFR-TKIs (e.g., sunitinib, sorafenib or pazopanib) may increase the rate of cardiotoxicity in metastatic settings. VEGF inhibitors modulate multiple signalling pathways; thus, the identification of the mechanism underlying cardiotoxicity appears challenging. VEGF signalling is vital for the maintenance of cardiomyocyte homeostasis and cardiac function; therefore, its inhibition can be responsible for the reported adverse effects. Disturbed growth factor signalling pathways may be associated with endothelial dysfunction, impaired revascularization, the development of dilated cardiomyopathy, cardiac hypertrophies and altered peripheral vascular load. Patients at high cardiovascular risk at baseline could benefit from clinical follow-up in the first 2–4 weeks after the introduction of targeted molecular therapy; however, there is no consensus concerning the surveillance strategy.

## 1. Introduction

Renal cell carcinoma (RCC) is among the most frequent malignant neoplasms of the kidney (present in 90% of cases) [1]. At the same time, RCC-related mortality is the highest among urological neoplasms. Since this type of tumour does not give easily recognizable alarming symptoms, in nearly one-third of patients it is diagnosed at the metastatic stage of the disease. The estimated overall 5-year survival is 76%; however, such survival is significantly reduced in patients with stage IV disease [2]. The treatment of this tumour is challenging since RCC comprises highly heterogeneous cancers with different underlying genetic and epigenetic mechanisms and molecular pathways, including clear cell (accounting for 70–75% of all cases and caused by the loss of tumour suppression gene VHL (von Hippel–Lindau) on the short arm of chromosome 3) and non-clear cell subtypes: papillary (15% of all kidney cancers), chromophobe RCC (occurring in 5–10% of all cases, typically a slowly growing type), collecting duct carcinoma and renal medullary carcinoma (aggressive types accounting for less than 5% of all RCC cases, resistant to most systemic treatment options), as well as sarcomatoid RCC (occurring in about 5% of all RCC cases, generally symptomatic and very aggressive) [3,4,5,6,7]. Approximately 20–50% of patients with localized disease will ultimately relapse and advance to the metastatic stage despite treatment [2,8]. Features of various subtypes of RCC are summarized in Table 1 (based on data from [9]).

The therapeutic options available for the treatment of advanced or metastatic RCC have changed over the years [3]. Initially, high-dose interleukin-2 [IL-2] and interferon-α were used to affect the immune system signalling cascade and intracellular oncogenic pathways. The introduction of molecular targeted agents and immunotherapies, including vascular endothelial growth factor receptor (VEGFR)-targeted molecules (e.g., tyrosine kinase inhibitors; TKI), immune checkpoint inhibitors (ICIs) and inhibitors of the mechanistic target of rapamycin (mTOR) has improved the prognosis and survival of patients with metastatic RCC (mRCC) [5,8,10]. Tyrosine kinases (TK) activate numerous proteins via phosphorylation, thus affecting signal transduction and regulating cellular activity [4]. In turn, TK inhibitors (TKI) attach to the ATP-binding pocket of these kinases, thus hampering their activity. Various VEGFR-TKIs proved to be effective in the treatment of patients with solid tumours [11]. Current guidelines suggest that the combination of two of the aforementioned drugs may prove most beneficial in the treatment of metastatic RCC [12,13,14]. However, the combination of two treatment options may increase the risk of toxicity in comparison to monotherapy. Cancer-treatment-related toxicities affect many organs; however, it appears that cardiovascular adverse effects are the most important since they worsen patients’ prognosis and quality of life [15]. Many studies and clinical trials have suggested that specific anti-VEGF tyrosine kinase inhibitors (TKI) such as sunitinib, sorafenib or pazopanib may possibly be responsible for a higher rate of cardiotoxicity in metastatic settings. Cardio-oncology is a relatively new area of knowledge which focuses on the treatment of cancer taking into consideration the risk of cardiovascular adverse effects of the therapies used [16]. This review will focus on the cardiotoxicity risk associated with VEGFR-TKIs treatment in patients with RCC.

We conducted a PubMed search to identify articles that are suitable for inclusion in this narrative review. We did not perform a systematic review. Terms that were searched for included: “cardiotoxicity”, “vascular endothelial growth factor receptor tyrosine kinase inhibitors”, “VEGFR-TKIs”, “renal cell carcinoma”, “RCC”, “prognosis”, “metastasis”.

## 2. Vascular Endothelial Growth Factor Receptor Tyrosine Kinase Inhibitors (VEGFR-TKIs)

Five tyrosine kinase inhibitors (TKIs) of the vascular endothelial growth factor receptor (VEGFR) pathway (VEGFR-TKI) have been approved by the Food and Drug Administration (FDA) in the treatment of metastatic RCC [17]. Pazopanib has been approved as the first-line treatment in patients with RCC3 [17]. These small-molecule tyrosine kinase inhibitors, including sorafenib, axitinib, sunitinib, cabozantinib and pazopanib, target vascular endothelial growth factor receptors (VEGFRs) [18]. VEGFR-TKIs act at the intracellular sites of the VEGF receptor, which affects the survival of microvascular endothelial cells [19]. Moreover, the less the VEGFR-TKI is specific, the more the effects are pronounced [20]. Inhibitors of VEGF signalling have been used with success in the treatment of various cancers since they prevent tumour angiogenesis, thus limiting its growth [17]. Inhibitors of VEGFR also enhance T lymphocyte trafficking into tumours, improving in consequence malignant cells’ responsiveness to immunotherapy [21,22]. The results of randomized phase III trials demonstrated their clinical benefits in the treatment of metastatic renal cell carcinoma [23]. The introduction of small-molecule targeted VEGFR-TKIs has increased median progression-free survival and overall survival in advanced/mRCC compared to previous treatment modes by 6 and 14 months, respectively [24,25]. Clinical data indicate that the use of TKIs is frequently associated with systemic adverse effects in patients. Nausea, diarrhoea, fatigue, rhabdomyolysis, hypertension, neutropenia, renal failure, QT prolongation and heart failure are among the frequent adverse effects [26]. Common adverse events related to therapy with sorafenib and sunitinib include hand-foot skin reaction, reversible skin rashes, haemorrhage, diarrhoea, leukopenia, increased pancreatic enzymes levels, hypophosphatemia and proteinuria [27,28]. Growing evidence suggests that despite being beneficial in terms of cancer, such treatment exerts cardiotoxic effects of VEGFRs-TKIs, including asymptomatic left ventricular (LV) dysfunction, hypertension and even congestive heart failure (CHF) [29,30].

Sunitinib, sorafenib, pazopanib and cabozantinib are used in the treatment of RCC. Sunitinib (known also as SU11248 or Sutent), an orally active multi-targeted receptor tyrosine kinase inhibitor, was approved by the FDA in 2006 for the treatment of, i.a., advanced renal-cell carcinoma (RCC), pancreatic cancer (PC), chronic myeloid leukaemia and imatinib-resistant gastrointestinal stromal tumour (GIST) [31,32]. This first-line therapy for metastatic RCC blocks VEGFR 1,2,3, platelet-derived growth factor (PDGF), colony stimulating factor-1, Fms-related receptor tyrosine kinase 3 (FLT-3) as well as tyrosine-protein kinase KIT (c-kit) [33,34]. Sunitinib’s actions involve the inhibition of angiogenesis and the limitation of blood supply to the tumour cells. The impact on blood supply, impairment of signal transduction, cellular metabolism and transcription is associated with elevated cardiovascular risk in cancer patients treated with such drugs [35]. Due to its relatively nonselective binding to the intracellular catalytic site of receptors, sunitinib inhibits a wide range of tyrosine kinases [36]. Its low specificity raised hope that it would inhibit angiogenesis and tumour growth but at the same time, it would be less vulnerable to drug resistance [37]. However, the inhibition of various growth factor pathways, particularly those involved in cardiac functioning, may be associated with cardiotoxicity. Sorafenib is a multikinase inhibitor affecting transmembrane VEGFR-2, VEGFR-3, FLT-3, PDGFR-B and KIT receptors, as well as intracellular serine/threonine-protein kinase B-raf (BRAF) and RAF proto-oncogene serine/threonine-protein kinase (CRAF) receptors, which is used in the treatment of patients with RCC [38]. The aforementioned kinases are involved in intracellular signalling pathways and angiogenesis; thus, their inhibition translates into hampered tumour growth [39]. A randomized, double-blind, placebo-controlled phase III trial (TARGET) enrolling over 900 patients previously resistant to therapy showed a significant benefit of sorafenib (vs. placebo) in terms of progression-free survival (PFS) and a 28% decrease in the risk of death in patients receiving sorafenib [40,41]. The National Comprehensive Cancer Network (NCCN) Task Force report [27] suggests that sorafenib may also be appropriate for certain naïve patients with clear cell mRCC. Pazopanib therapy is associated with the inhibition of VEGFR 1–3 and subsequent hampering of angiogenesis and RCC regression [42]. This drug also hinders the actions of stem cell factor KIT receptors and platelet-derived growth factor receptors. Finally, cabozantinib is an oral TKI of MET, VEGFRs and Anexelekto (AXL). This therapy was used in the phase III METEOR trial in pre-treated patients with advanced RCC. It was found to increase progression-free survival (PFS) and objective response rate improvements [43,44]. Currently, cabozantinib has received the approval of the FDA for RCC [45].

## 3. Cardiotoxicity and Involved Mechanisms

Therapy with VEGFR inhibitors has been demonstrated to improve overall survival and progression-free survival (PFS) in patients with metastatic renal cell cancer. However, tyrosine kinase inhibitors (VEGFR-TKIs) can induce adverse cardiovascular (CV) toxicities [25,40,46]. Many pathways inhibited by tyrosine kinase inhibitors play crucial roles in the preservation of cardiovascular development, CV function and response to CV stress [47,48,49]. Such therapies interfere with key cardiovascular signalling pathways; thus, they may induce considerable cardiovascular toxicities [50].

The term cardiotoxicity refers to cardiovascular complications of therapies which result in higher morbidity and mortality [51]. The occurrence of this phenomenon differs between oncological therapies, some of which are associated with early clinical manifestation of cardiotoxicity, while in the case of others the adverse effects appear years after the initiation of treatment. Since patients with cancers are administered many drugs, the prediction of cardiotoxicity seems challenging. According to estimations, the incidence of VEGFR-TKI cardiotoxicity is in the range of 3–30% and depends on the drug, study population and diagnostic criteria [48,51,52,53]. Cancer survivors suffer from late CV risk due to prior cardiotoxic exposure and the appearance of new cardiovascular risk factors with advancing age. Cardiovascular mortality rates in paediatric cancer survivors have been found to be up to ten times higher compared to age-matched controls [54]. Late CV risk is also considerably higher among long-term adult cancer survivors and the risk is particularly pronounced in adult-onset cancer survivors with underlying CV risk factors [55]. Therefore, it is important to recognise potential long-term CV toxicity in cancer survivors in order to implement aggressive correction of CV risk factors in this population and treatment of incident CV dysfunction [56].

### 3.1. Possible Mechanisms

Since VEGF inhibitors affect multiple signalling pathways, the identification of the underlying mechanism that causes cardiotoxicity can be challenging [51]. Cardiotoxicity may be ascribed to the inhibition of tyrosine kinases that are normally expressed in non-neoplastic tissues, including blood vessels and the myocardium; however, the exact mechanism remains not fully understood. VEGF signalling is of importance for the maintenance of cardiomyocyte homeostasis and cardiac function; therefore its inhibition, especially accompanied by the blockade of PDGFR, RSK and AMPK kinases participating in cardiomyocytes energy metabolism and survival, can be responsible for the observed adverse effects [46].

The results of studies have demonstrated that disturbances related to these growth factors may be associated with endothelial dysfunction, impaired revascularization, the development of dilated cardiomyopathy, cardiac hypertrophies and altered peripheral vascular load [57,58,59,60,61]. The family of VEGFR (VEGFR-1, -2 and -3) is involved in numerous vascular functions important for the preservation of proper functioning of the cardiovascular system [36,62,63]. Since VEGF promotes endothelial cell proliferation and survival, thus contributing to vascular integrity, the inhibition of VEGF signalling is associated with a diminished regenerative capacity of endothelial cells, enhanced proliferation of vascular smooth muscle cells, increased haematocrit and blood viscosity promoting pro-coagulant changes and favouring thrombosis [27,64]. The loss of VEGF signalling has been suggested to cause oxidative stress, vascular rarefaction, the inhibition of nitric oxide pathway and glomerular injury and result in the development of hypertension [65]. Moreover, VEGF inhibition can induce renal thrombotic microangiopathy [66]. Available data indicate that the sequestration of VEGF results in impaired adaptive cardiac hypertrophy in response to pressure overload [59].

The evidence concerning the cardiovascular toxicity of sunitinib appears to be most convincing [29,67,68]. The majority of data concerning the mechanisms of sunitinib-induced cardiotoxicity come from animal studies. Sunitinib-related cardiac side effects are associated with coronary microvascular dysfunction, the triggering of the endothelin-1 system, the inhibition of adenosine 5‘-monophosphate-activated protein kinase (AMPK) resulting in the impairment of normal mitochondrial function and consequent cellular energy homeostasis compromise within the heart, as well as hindering of mast/stem cell growth factor receptor [36]. Indeed, the study of sunitinib’s influence on cardiac mitochondrial function in the culture of cardiomyocytes revealed considerable abnormalities in mitochondrial structure [29]. This finding was confirmed in another study in which the incubation of rat neonatal cardiomyocytes with a high dose of sunitinib triggered the activation of a caspase-9-related mitochondrial apoptotic pathway as well as the loss of mitochondrial membrane potential and energy rundown as a result of the inhibition of AMP-activated protein kinase [69]. The impairment of mitochondria was presented also in other animal studies. The administration of 40 mg/kg per day of sunitinib for 12 days to mice was associated with the appearance of aberrantly shaped and swollen mitochondria with disrupted cristae [29]. However, cardiomyocyte apoptosis was not triggered until severe hypertension was induced with the use of phenylephrine in the studied animals. The occurrence of cardiac apoptosis was seven times higher in mice treated with sunitinib (10 mg/kg per day) + phenylephrine in comparison to animals fed only with phenylephrine. Thus, it seems that sunitinib-related mitochondrial dysfunction and apoptosis are facilitated by the presence of additional cardiac stress [36]. Since AMPK is involved in the maintenance of cardiac energy homeostasis in a state of enhanced cardiac stress, the impairment of this pathway by sunitinib may result in cardiac dysfunction. AMPK may participate in the hindering of anabolic pathways and inducing energy generation under energy stress via the regulation of acetyl-CoA-carboxylase (ACC) activity and subsequent uptake and metabolism of prime cardiomyocytes energy source-free fatty acids. The role of altered AMPK signalling in heart failure was demonstrated for the first time in patients with the familial form of hypertrophic cardiomyopathy, a rare disease associated with missense SNP within γ2 regulatory subunit of AMPK (PRKAG2) [70]. The mechanism of AMPK-inhibition-related sunitinib-induced cardiotoxicity under stress conditions is slowly emerging, but the picture is still not complete. It appears that AICAR (5-aminoimidazole-4-carboxamide-1-β-D-ribofuranoside; acadesine) may be involved in this process. According to studies, AICAR, which activates AMPK, may diminish myocardial ischemic injury via the limitation of oxidative stress, leukocyte plugging and platelet aggregation [71]. However, the blockage of AMPK was found to reverse the beneficial effects of acadesine on the apoptosis of rat cardiomyocytes exposed to hypoxic stress [72]. Also, the role of AMPK signalling in the settings of pressure overload was assessed. A study of AMPKα2 knockout mice (lacking the catalytic unit of AMPK expressed predominantly in the heart) which underwent transverse aortic constriction (TAC) showed a more pronounced loss of LV function, accelerated LV hypertrophy and considerably increased mortality in comparison to wild-type animals after 3 weeks of TAC. In an animal model, the administration of sunitinib reduced the phosphorylation of acetyl-CoA-carboxylase (ACC) in heart tissue which translated into the loss of the activity of AMPK [69]. In turn, the delivery of constitutively active AMPK into cardiomyocytes was associated with their partial resistance to sunitinib-triggered apoptosis. These findings may suggest that the impairment of AMPK signalling may be associated with disturbed adaptation to systolic pressure overload and consequent severe cardiac dysfunction. This mechanism may be potentially responsible for cardiotoxicity observed in hypertensive patients treated with sunitinib. However, in vitro study of sunitinib’s effect on isolated rat heart mitochondria and intact rat myoblast cells failed to demonstrate the direct adverse impact of treatment on mitochondrial function [73]. According to the authors, sunitinib-induced mitochondrial abnormalities are due to the inhibition of RSK, which can stimulate proapoptotic factor Bad, leading to the release of cytochrome and apoptosis. Another study confirmed that sunitinib acts as a strong inhibitor of RSK [74].

Also, the impact of sunitinib on platelet-derived growth factor receptor (PDGFR) and AMP-activated protein kinase (AMPK) may affect cardiomyocyte function and survival [46,75]. Some authors have hypothesized that apart from the greater release of endothelin-1, lower production of nitric oxide in the arteriole wall as well as microvascular rarefaction (involving the apoptosis of endothelial cells and the remodelling of capillary beds) could also be responsible for VEGF-inhibitor-related hypertension [76,77,78]. The observed rise in resistive load may support the role of the reduced number of microvessels in the development of sunitinib-related hypertension [50]. The results of other studies also demonstrated that TKI-induced hypertension may be associated with greater systemic afterload following VEGF inhibition as well as the destruction of endothelial cells, VEGF-receptor-inhibition-related disturbances in vasoconstrictor–vasodilator balance, lower survival of mesangial cells and impaired glomerular function and filtration [79,80,81,82]. Moreover, Catino et al. [50] suggested that the worsening of arterial stiffness may also contribute to the pathophysiology of hypertension induced by sunitinib. The authors suggested that the combination of calcium channel blockers with inorganic nitrates may prove useful in the management of hypertension in sunitinib-treated patients due to their vasodilating properties and ability to ameliorate conduit artery function, which appears to be impaired in this group of patients [50]. The aforementioned increased arterial stiffness is also an important risk factor for coronary and cerebrovascular disease [29,50].

The cardiotoxicity of sorafenib has been suggested to be related to the imbalance of pro-survival factor RAF1 (probably also involved in cardiac functioning) and pro-apoptotic factors MST2 (serine/threonine kinase 3) and ASK1 (apoptosis signal-regulating kinase 1) [46]. RAF1 was suggested to inhibit apoptosis-signal-regulating kinase 1 (ASK1) and mammalian sterile 20 kinase 2 (MST2), which exert apoptotic, ERK-independent effects and participate in oxidant-stress-induced injury [46]. Sorafenib-induced impairment of RAF1–ASK1 and/or RAF1–MST2 interactions may cause higher cardiotoxicity compared to solely ERK cascade hindering [46]. A study in an animal model demonstrated that a cardiac-muscle-specific Raf-1-knockout (Raf CKO) mirroring Raf-1 inhibition caused LV systolic dysfunction and heart dilatation induced by considerable elevation of apoptotic cardiomyocyte amounts and the promotion of fibrosis [83]. TKI drugs may also impair angiogenesis by affecting the Src family and downstream RAF1 [84]. According to studies, TKI could target the c-kit, thus negatively affecting the expression of AT2 (angiotensin II receptor type 2) involved in the repair of ischemic injury [85]. However, a vast range of cardiotoxic phenotypes and degrees of toxicity cannot be ascribed only to the above-mentioned mechanisms. According to another theory, this class of drugs may exert a cardiotoxic effect via acting “off-target” [20]. It has also been suggested that TKI may trigger mitochondrial toxicity, thus damaging metabolically active cardiomyocytes of the heart [46]. Will et al. [73] provided evidence that sorafenib may act as an inhibitor of Complex V and mitochondrial uncoupler. The results of an animal (rat) study revealed that sorafenib disturbed mitochondrial cristae [86]. In turn, studies of cell cultures indicated that treatment of cancerous cell lines with sorafenib increased the levels of the autophagy markers Beclin1 and LC3 (microtubule-associated protein 1 light chain 3) [87]. In general, autophagy is considered a protective mechanism; low levels of autophagy in hearts appear to maintain the homeostasis and turnover of organelles; however, its enhanced activation may result in cellular death [88,89,90]. Also, apoptosis has been suggested to contribute to cardiomyocyte loss and subsequent heart failure or hypertensive cardiomyopathy [91,92]. The activation of the pro-apoptotic protein BAD is associated with the downregulation of the anti-apoptotic protein Bcl-2’s expression and the subsequent triggering of the initiator and effector caspases, caspase-9 and -3, and the promotion of death of cardiomyocytes [93]. Moreover, according to some authors, the cardiotoxic effects of sorafenib are associated with an increase in metabolites such as urea and fatty acid levels in plasma [94].

**Figure 1:**
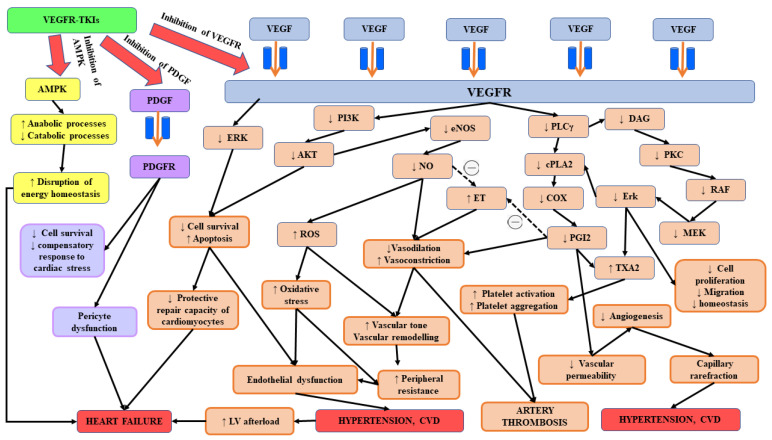
The results of VEGFR, AMPK and PDGFR inhibition leading to cardiotoxicity of VEGF signalling inhibition.

Another theory concerning cardiotoxicity related to VEGF inhibitors states that perfusion–contraction match may play a role in this phenomenon [95,96]. According to this thesis, these drugs may contribute to the decrease in myocardial perfusion that is associated with the additive effects of diffuse, non-significant reductions in the coronary arteries’ luminal diameter [97]. Evolving aberrations of the coronary microcirculation may result in the impairment of myocardial perfusion. Since patients with cardiovascular diseases display a lower tolerability margin in this area, cardiotoxicity in this group is more common [98]. Chintalgattu et al. [99] confirmed that sunitinib can diminish coronary flow reserve, impair the integrity of the coronary microcirculation and worsen cardiac function. They suggested that the inhibition of the PDGF signalling pathway could be responsible for these effects via a reduction in the pericyte population, resulting in the destabilization of endothelial cells, coronary microcirculation and eventually cardiac function. In the opinion of the authors, systemic hypertension combined with impaired PDGFR signalling could be responsible for the development of heart failure in patients treated with sunitinib [100]. PDGF is a vital growth factor for various cell types, such as cardiomyocytes, smooth muscle cells, endothelial cells and stromal cells, that promote angiogenesis and the maintenance of endothelial function [46,101]. It was also demonstrated to mediate the signalling between heart myocytes and adjacent endothelial cells [102]. Both receptor subtypes of PDGF are inhibited by sunitinib. The inhibition of PDGFR receptor-ß tyrosine kinase by sunitinib is associated with decreased myocardial pericytes, myocardial microvascular density and worsened cardiac function [99]. The inhibition of PDGFR signalling has been found to adversely affect cardiac function, particularly in the stressed heart [36]. The aforementioned pericyte–endothelial–myocardial coupling appears to partly explain the role of VEGF signalling inhibition in cardiotoxicity [103]. Suggested mechanisms of cardiotoxicity of VEGF signalling inhibition are presented in Figure 1.

### 3.2. The Results of the Studies

Clinical data show that VEGF inhibitors, especially TKI, can trigger either reversible or irreversible cardiac side effects [51]. According to estimations, heart dysfunction is observed in 3–15% of patients treated with sunitinib, pazopanib and axitinib, while symptomatic heart failure occurs in 1–10% of these patients [104,105,106]. However, clinical data from large clinical trials are not available for sorafenib and vandetanib, both of which can also promote cardiac dysfunction. VEGF-inhibitor therapy-related hypertension develops in approximately 19 to 47% of patients [76]. Meta-analyses of randomized clinical trials of sorafenib, pazopanib and sunitinib demonstrated that such treatment is associated with a higher risk of decline in left ventricular ejection fraction (LVEF), hypertension and arterial thrombotic events [66,107,108,109,110,111]. In turn, a recent meta-analysis assessing treatment with sunitinib, axitinib, ponatinib, vandetanib, cabozantinib, sorafenib, pazopanib and regorafenib demonstrated a 2.69-fold rise in the risk of congestive HF (all grades) [52,53]. Therapy with either sorafenib or sunitinib was found to be associated with hypertension, LV systolic and diastolic dysfunction, heart failure (HF) and myocardial ischemia [33,47,48,67]. Sorafenib therapy appeared to affect resistive and pulsatile load [112]. Other clinical events associated with cardiotoxicity include congestive heart failure (CHF) and arterial thromboembolic events (ATE) [27,113,114]. Moreover, adjuvant sunitinib and sorafenib could trigger arrhythmia and cardiac ischemia [33]. Abdel-Qadir et al. [115] demonstrated a higher risk of arterial thromboembolism (odds ratio [OR]  =  1.52, 95% confidence interval (CI) 1.17–1.98) in patients treated with VEGF inhibitors. The results of another meta-analysis revealed a considerably greater risk of all-grade bleeding, all-grade and high-grade hypertension as well as all-grade cardiac dysfunction in patients with tumours receiving VEGFR-TKIs [116]. Also, other studies indicated a significantly greater risk of high-grade (RR 4.60, 95% CI 3.92–5.40) and all-grade (RR 3.85, 95% CI 3.37–4.40) hypertension [108]. VEGFR-TKI therapy was also suggested to trigger QTc interval prolongation [117]. The most pronounced effect was reported in the case of sunitinib and vandetanib. Bayesian network meta-analysis of nine FDA-approved VEGFR-TKIs demonstrated their impact on the occurrence of all grades and grade 3 or higher cardiovascular events, hypertension and cardiac damage [11]. Totzeck et al. [118] found a higher relative risk of cardiac ischemia (RR 1.69, 95% CI 1.12–2.57), LV systolic dysfunction (RR 2.53, 95% CI 1.79–3.57) QT corrected interval prolongation (RR 6.25, 95% CI 3.44–11.38) and arterial hypertension (RR 3.78, 95% CI 3.15–4.54), especially in sunitinib-treated patients. However, a re-analysis of the aforementioned meta-analysis including 71 trials and eight different VEGFR-TKIs suggested that the previously observed effect was exaggerated since, according to the authors, such treatment was associated with a slight elevation in the risk of bleeding, hypertension, thrombocytopenia and arterial thrombotic damage [119].

The results of a recent meta-analysis indicated that the incidence of sunitinib-induced HF might amount to 4.1%, while the prevalence of asymptomatic LVEF deterioration could be even higher in patients with metastatic disease [113,120]. The worsening of LV ejection fraction appears usually in the first cycle of treatment with sunitinib [50]. A multi-centre, longitudinal prospective cohort study confirmed early worsening of vascular function following sunitinib exposure [50]. Such treatment was associated with higher markers of resistive load, total peripheral resistance and arterial elastance. Catino et al. [50] reported the worsening of diastolic dysfunction (E/e’) and LV filling pressures (BNP) in sunitinib-treated patients. Sunitinib’s impact on cardiac function was reported in a phase III trial, in which either sunitinib or interferon alfa was used in the treatment of patients with advanced RCC [25]. In that trial, the sunitinib-related decline in left ventricular ejection fraction (LVEF) was observed in 10% of patients, including 2% of subjects who developed a grade 3 decline in LVEF. The discontinuation of treatment or dose reduction appeared to reverse this condition. A retrospective study enrolling patients with RCC or imatinib-resistant gastrointestinal stromal tumours (GISTs) demonstrated symptomatic grade 3 or 4 LV dysfunction in 15% of sunitinib-treated patients [48]. The results of other studies have suggested a higher, up to 27%, incidence of cardiac problems [121]. Based on the results of a prospective study, Narayan et al. [68] suggested that the declines in LVEF occurring in approximately 9.7% of patients, even the substantial ones observed in 1.9% of patients, returned to near baseline values despite the continuation of sunitinib therapy in a reduced dose when careful cardiovascular management was provided. Moreover, according to the authors, routine cardiac monitoring in asymptomatic individuals after the third cycle of therapy will not bring great clinical benefit since at that time cardiac dysfunction rates are low.

A meta-analysis of 16 clinical trials including 6935 patients provided evidence for a higher risk of congestive heart disease in patients treated with sunitinib (risk ratio 1.81; 95% CI 1.30–2.50; *p* < 0.001). The 1.5% incidence of high-grade CHF in this analysis translated into a three-times-increased risk of developing serious cardiovascular events (risk ratio 3.30; 95% CI 1.29–8.45; *p* < 0.01). Chu et al. [29] indicated that patients treated with sunitinib experience continuous, gradual worsening of cardiac function. This effect is also observed in many patients with stabilized hypertension dynamics (BP < 140/90 mmHg) treated with beta-blocker and angiotensin-converting enzyme inhibitors. The authors suggested that perhaps more aggressive BP control may prove beneficial. Increased resting systemic blood pressure and higher systemic and coronary vascular resistance were also reported in animals treated with sunitinib [122]. Chronically elevated afterload led to hypertrophic heart remodelling. One animal study indicated that the process of cardiomyocyte apoptosis occurred as a result of the combined effect of sunitinib treatment and the increase in blood pressure [29]. The results of retrospective studies have found that the incidence of sunitinib-induced cardiotoxicity in mRCC patients ranges from 3% to 30% [47,48,52,123]. Such discrepancies in results could stem from the fact that cardiac monitoring protocols are not standardized and different definitions of cardiotoxicity were used in studies [68].

According to one of the studies, sorafenib also markedly decreased left ventricular (LV) pressure, indexes of myocardial contractility and relaxation as well as prolonged systolic and diastolic periods [94]. Such therapy was also found to trigger vasospasm [124]. Sorafenib-related elevation in mid to late systolic load of the LV can potentially lead to myocardial hypertrophy, fibrosis and heart failure [125,126,127,128]. In turn, one of the most common side effects of cabozantinib is the development of hypertension with an incidence of 37% in the METEOR trial (15% of grade 3–4) and 81% in the CABOSUN study (28% of grade 3–4) [43,44,129,130]. Such a high frequency of hypertension related to cabozantinib treatment was confirmed in a meta-analysis. Compared to other VEGFR-TKIs, the occurrence of such adverse effects was considerably higher in the case of cabozantinib [129]. A prospective study focusing on the chances of LV systolic dysfunction in cabozantinib-treatment patients revealed an extremely modest risk of developing this disorder [18]. A decline in LVEF by more than 10% was observed in 11.1% of cases after 3 months of therapy; however, it did not translate into LV systolic dysfunction or the appearance of clinical symptoms. This mode of cancer management was not associated with an elevation in cardiac biomarkers, such as proBNP or hsTnI. Iacovelli et al. [18] reported that cabozantinib did not significantly increase the risk of cardiac dysfunction even in patients with cardiovascular comorbidities.

Also, the use of pazopanib has been reported to be associated with cardiotoxicity in the form of hypertension (HTN), thrombotic events, heart failure (HF) with a reduced left ventricular ejection fraction (LVEF) and myocardial ischemia in many patients [52,131,132]. The incidence of hypertension reaches up to 40% and an elevation of N-terminal B-type natriuretic peptide (NT-pro-BNP) levels is observed in up to 26% of patients while HF is reported in 2.4% [52,53]. Another meta-analysis revealed a slightly higher incidence of all-grade HF (3.2%) related to therapy with VEGFR-TKIs [106]. The cardiotoxicity of pazopanib was confirmed in animal studies. One of them demonstrated that the administration of pazopanib considerably increased blood pressure (BP) and diminished CO. The latter finding may suggest early cardiomyocyte stress and probable remodelling in the presence of higher mean arterial pressure [17]. However, another study demonstrated that pazopanib did not negatively affect mouse growth or survival [133].

In contrast to the aforementioned studies, the results of other research have demonstrated a very low risk of cardiac ischemia/infarction related to treatment with angiogenesis inhibitors or TKIs [38,67,114,134]. It reached 2.9% in patients on sorafenib therapy, 1.5% in those on bevacizumab therapy and <1.0% in patients treated with sunitinib. A meta-analysis of the impact of pazopanib, sunitinib, sorafenib and vandetanib on the occurrence of venous thromboembolic events failed to demonstrate a significant relationship [109]. Also, a meta-analysis concerning the risk of arterial thromboembolic events (ATE) and VTEs associated with various VEGFR-TKIs demonstrated a lack of marked increase in the risk of developing all-grade and high-grade VTEs [135]. It seems that a higher occurrence of cardiotoxicity may be associated with a relatively high presence of pre-existing CV disease and/or cardiovascular risk factors in RCC patients [136]. Numerous studies have indicated that hypertension and cardiovascular disease at baseline are crucial predictors of major adverse cardiac events (congestive heart failure, cardiovascular death, myocardial infarction) after the VEGF-TKI sunitinib therapy [29,98,123]. Also, the prevalence of sorafenib-related LVEF dysfunction and/or CHF is higher in patients with a history of hypertension or coronary artery disease [137,138,139]. More than 70% of hypertensive patients (grade 3 HTN) developed LV systolic dysfunction following the initiation of sunitinib treatment. Khakoo et al. [47] hypothesized that acute rises in blood pressure together with the adverse impact of sunitinib on cardiomyocytes may result in the impairment of cardiac response to BP elevation and subsequent heart failure. The randomized, double-blinded phase III ECOG 2805 trial of adjuvant sunitinib, sorafenib or placebo demonstrated a low incidence of treatment-related significant LVEF decline in formerly untreated patients with completely resected RCC at high risk for recurrence and without baseline cardiovascular comorbidities. The authors suggested that the prevalence of cardiac dysfunction could be higher in the population of symptomatic patients. Therefore, it appears there is a need for close CV monitoring combined with immediate hypertension therapy in RCC patients treated with sunitinib or sorafenib [33]. The control of hypertension can potentially decrease the risk of heart failure. In turn, appropriate HF management can ameliorate already-developed cardiac dysfunction [140].

The occurrence of cardiotoxicity could be underestimated in some studies since many clinical trials exclude patients with cardiovascular diseases [141]. A real-life setting study of major adverse cardiovascular events (MACE) incidence in patients treated with TKI revealed that arterial thrombotic events occurred in 3.99% of study participants, rhythm disorders (atrial fibrillation, atrioventricular block) in 2.66%, and pulmonary embolism and heart failure in 1.57% at 1 year of follow-up [141]. According to the authors, a high incidence of atrial fibrillation in the early period of therapy was associated with an anti-VEGFR treatment-related increase in blood pressure and diastolic dysfunction of the left ventricle. After the initial period, AF could be associated with early remodelling of the atrial ventricles.

According to the recommendations of the European Society for Medical Oncology echocardiography at baseline and every 3 months for the first 6 months and optionally the evaluation of global longitudinal strain should be performed during the first months of TKI therapy in order to carefully monitor arrhythmias, the development of heart failure and pulmonary embolism [141,142]. Considering the increasing prevalence of thrombotic events, high-risk patients should obtain adequate therapy to prevent thrombotic adverse effects [141]. Patients at high cardiovascular risk at baseline could benefit from clinical follow-up in the first 2–4 weeks after the introduction of targeted molecular therapy with, e.g., sorafenib, sunitinib or pazopanib [51]. Patients should undergo periodic reassessment of cardiac function to detect early symptoms of developing cardiac complications. There is no consensus concerning the surveillance strategy. It seems rational to perform periodic echocardiography until the stabilization of LVEF values. Moreover, the determination of values of cardiac biomarkers, such as troponin or N-terminal pro-B-type natriuretic peptide (NT-proBNP), is recommended to increase the number of diagnosed complications. The timing of the aforementioned assessments should be adjusted to the needs of a given patient, his baseline cardiovascular risk and antitumour regimen [143]. The lack of optimal surveillance negatively affects clinical outcomes. However, the optimal timing of biomarker testing has still not been established. One systematic review suggested that the use of ACE inhibitors, angiotensin II receptor blockers (ARBs) and beta-blockers appears beneficial in patients who have developed develop asymptomatic LV dysfunction or HF during the treatment of cancers [144]. The results of studies presenting benefits and cardiotoxicity risk related to VEGF-TKI are presented in Table 2. 

The potential risk of cardiotoxicity of TKI has resulted in the addition of special warnings on product labelling [93]. However, sunitinib remains the gold standard in the treatment of some tumours despite the current focus on its cardiotoxicity. Many studies have demonstrated its effectiveness and safety and it seems that a better understanding of the underlying mechanisms would enable the reduction of this risk [32,151]. The inclusion of studies in this narrative review lacked a systematic approach which could affect our conclusions concerning this field.

## Figures and Tables

**Table 1:** Summary of features of various subtypes of RCC.

Features	Clear Cell RCC (ccRCC)	Papillary RCC (pRCC)	Chromophobe RCC (cRCC)	Clear Cell Papillary RCC (ccpRCC)	Multilocular Cystic *
Occurrence	~70–75% of RCC	~10–20% of RCC	~5–7% of RCC	1–4% of RCC	~1–4% of RCC
Age of onset	>50 years	>50 years	>50 years		40–50 years
Morphology	Clear or pale appearance, sometimes yellow Acinar, nested, alveolar, tubular architecture with small cysts Capillary vessels intimately associated with the tumour	Cells with finger-like projections. Papillary architecture, tubulo-papillary, glomeruloid, and dense papillary simulating solid areas also occur Slender papillae lined with a single layer of cuboidal cells with scant, basophilic cytoplasm and inconspicuous nucleoli	Solid architecture with extension to adjacent renal parenchymal entrapping pre-existent tubules Large eosinophilic cells and vegetal-like cells with distinct cytoplasmic membrane. Eosinophilic or finely granular cytoplasm with perinuclear halos and nuclei showing “raisinoid” morphology	Cells with “piano-key-like” pattern Clear cells with tubular (predominant), papillary, acinar, cystic, cords and solid growth	Cysts lined with cells with clear cytoplasm and low-grade nuclei with variable sizes separated by delicate septae that may contain cords of clear cell
Characteristic traits	Originates from proximal nephron/ tubular epithelium Aggressiveness related to the stage and Furham grade	2 types Originates from distal nephron/tubular epithelium typically low grade; cases with high-grade nuclear features (prominent nucleoli, non-basophilic cytoplasm), sometimes invasive growth	Originates from distal nephron/intercalated cells of distal tubules Less aggressive compared to ccRCC and pRCC (mortality ~10% patients) Rhabdoid/sarcomatoid and tumour necrosis—more aggressive behaviour	Indolent, not metastatic	More prevalent in males Indolent Not metastatic Good prognosis
**Features**	**Medullary Carcinoma**	**Mucinous Tubular and Spindle Cell Carcinoma**	**Collecting Duct Carcinoma (CDC)**	**Xp11 Translocation RCC**	**Unclassified RCC**
Occurrence	~1% of RCC	Rare	~1–2% of RCC		3–6% of RCC
Age of onset	20–30 years	40–50 years	20–30 years	childhood	Variable
Morphology	Similar to CDC, tubular, papillary and infiltrative architectures Adenoid cystic, reticular and microcystic patterns	Tightly packed tubular component lined with cuboidal cells transitioning into a bland spindle cell component Variable amount of mucinous/myxoid stroma Low-grade tubules, spindle cell component and mucin	Predominant tubular morphology (tubulo-papillary and papillary patterns are also common) Desmoplastic stromal reaction High-grade nuclei Absence of other RCC subtypes	Papillae lined with epithelioid clear and eosinophilic cells with abundant psammoma bodies	More than one cell type visible under a microscope
Characteristic traits	Originates from distal nephron Extremely aggressive centred in renal medulla Associated with sickle cell trait/disease/related hemoglobinopathies	Originates from distal nephron/tubular cells Indolent, usually low-grade; rarely high-grade nuclei and sarcomatoid, rarely metastatic	Originates from distal nephron High-grade adenocarcinoma Medullary involvement High aggressiveness (2-year mortality 70%)	Overall prognosis comparable with clear cell RCC	Aggressive High mortality

* multilocular cystic renal neoplasm of low malignant potential.

**Table 2:** The results of studies presenting benefits and cardiotoxicity risk related to VEGF TKIs.

Name of Drug/Mechanism of Action	Anti-Tumour Effects	Possible Cardiotoxicity
**Sorafenib** Inhibitor of VEGFR-2, -3, FLT-3, PDGFR-B and KIT, BRAF and CRAF receptors	Hampers tumour growth [38]. 28% reduction in the risk of death among patients receiving a dose of 400 mg twice daily compared with placebo. Median progression-free survival of 5.5 mo compared to 2.8 mo in the placebo group (HR 0.44; 95% CI 0.35 to 0.55; *p* < 0.01). PFS shorter in second- vs. first-line treatment [145]. In phase 1 clinical studies: sorafenib used in advanced, refractory solid tumours demonstrated disease stabilization with acceptable toxicity [146]. A phase 2 randomized discontinuation trial of metastatic RCC: tumour shrinkage, 50% of patients were progression free vs. 18% on placebo, significantly increased PFS (24 weeks vs. 6 weeks on placebo; *p* = 0.0087) [147]. Phase 3 TARGET trial (ccRCC): interim analysis: median PFS: 5.5 vs. 2.8 mo on placebo, *p* < 0.001) [40]. No statistically significant difference in OS between study arms. Survival advantage over placebo when patients crossing over were censored (17.8 vs. 14.3 months, respectively; *p* = 0.029) [41].	Hypertension occurred in 12% of patients receiving 400 mg twice daily [105]. Significantly increased risk of all-grade hypertension with RR of 6.11 (2.44–15.32], *p* < 0.001) compared with controls [66]. In meta-analysis, RR of ATEs associated with sorafenib and sunitinib was 3.03 (95% CI, 1.25 to 7.37; *p* = 0.015) compared with control patients [107]. Increased risk of all-grade hypertension (RR 1.99; 95% CI 1.73–2.29) and high-grade hypertension (RR 0.98; 95% CI 0.75–1.30 [111]. LVEF decline >15% from baseline and below the institutional lower limit of normal reported in 1.4% of patients [33]. Increase by ≥10 mmHg in SBP in 75% of patients and by ≥20 mmHg in 60% of patients from baseline value; mean change of 20.6 mmHg (*p* < 0.0001) after 3 weeks of therapy [112]. Cardiac ischemia or infarction occurred in 3% of patients receiving a dose of 400 mg twice daily in 6-week cycles for the first 24 weeks and in 8-week cycles thereafter [40].
**Sunitinib** Blocks VEGFR 1, 2, 3, PDGF, CSF-1, FLT-3, c-KIT	Inhibition of angiogenesis [37]. Limitation of blood supply to the tumour cells [37]. Longer overall survival compared with IFN-α. Increases progression-free survival in the first-line treatment of patients with metastatic RCC vs. INF-α (randomized, phase III trial) [24]. Increases progression-free survival (11 mo vs. 5 mo INFα). Improves objective response rate (31% vs. 6% INFα, *p* < 0.001). Improves quality of life compared to INFα (*p* < 0.001) [25].	Cardiac dysfunction occurred in 11% of patients receiving once-daily dose of 50 mg for 4 weeks, followed by 2 weeks without treatment [104]. Myocardial infarction or ischemia occurred in 4% of patients [104]. In meta-analysis, RR of ATEs associated with sorafenib and sunitinib was 3.03 (95% CI, 1.25 to 7.37; *p* = 0.015) compared with control patients [107]. Increased risk of all-grade hypertension (RR 2.20; 95% CI 1.92–2.52) and high-grade hypertension (RR 0.81; 95% CI 0.62–1.06) [111]. LVEF decline >15% from baseline and below the institutional lower limit of normal reported in 1.8% of patients [33]. 2.7% of patients receiving sunitinib malate developed HF which resulted in substantial morbidity and mortality. Symptomatic HF occurred after a mean of 22 days from treatment initiation. Decline in cardiac function and elevations in BP pressure were not completely reversible [47]. 15% of patients developed symptomatic grade 3/4 HF [48]. Increased mean SBP by 9.5 mm Hg (95% Cl 2.0–17.1; *p* = 0.02) and DBP by 7.2 mm Hg (95% CI 4.3–10.0; *p* < 0.001) in all participants. Increased large-artery stiffness and resistive load (*p* < 0.05) and changes in pulsatile load [50].
**Pazopanib** Targets VEGFR-1, -2, -3, PDGFR-α and -β, c-KIT	Inhibitor of angiogenesis and RCC regression [41]. PFS of 10.6. Median OS of 14.5 mo [148]. PARACHUTE, phase IV trial: 39% of patients remained progression free (at 12 months); median PFS was 10 months (95% CI: 8.48–11.83) [149]. 19% of patients were long-term responders. CR/PR in 24%, stable disease in 44% and PD in 31% patients [149]. Phase III COMPARZ study: Median time to response—11.9 weeks, CR/PR ≥ 10 months in 14% of patients, PFS ≥ 10 months—31% [150].	*Animal study* 30 mg/kg of pazopanib twice daily—significant elevation in blood pressure after 2 weeks which persists for the duration of dosing [17]. Decrease in CO suggestive of early cardiomyocyte stress and possible remodelling [17]. *Human trial* Hypertension—one of most frequent AE Cardiac dysfunction present in 13% of patients treated with once-daily dose of 800 mg (continuous dosing) [104]. Myocardial infarction or ischemia occurred in 2% of patients [104]. Increased risk of developing all-grade (RR 4.97, 95% CI 3.38–7.30, *p* < 0.001) and high-grade hypertension (RR 2.87, 95% CI 1.16–7.12, *p* = 0.023) [111].
**Cabozantinib** Inhibitor of c-MET, VEGFR2, Ret, KIT, FLT-1/3/4, Tie2, AXL	Increased PFS compared with sunitinib in CABOSUN trial (median of 8.6 vs. 5.3 mo; HR 0.48, 95% CI 0.31 to 0.74; two-sided *p* = 0.0008) [42]. Higher median OS of 26.6 mo for cabozantinib vs. 21.2 mo for sunitinib (HR 0.79, 95% CI 0.53 to 1.2; two-sided *p* = 0.27) [42]. Overall survival of 21.4 months (95% CI 18.7–not estimable) vs. 16.5 months (14.7–18.8) with everolimus (HR 0·66 [95% CI 0.53–0.83]; *p* = 0.00026) [44]. Improved PFS (HR 0.51 [95% CI 0.41–0.62]; *p* < 0.0001) and objective response (17% [13,14,15,16,17,18,19,20,21,22] [44].	Hypertension occurred in 28% of patients receiving dose of 60 mg once per day [42]. Hypertension as the most common grade 3 or 4 adverse event in 15% of patients. Significantly increased risk of developing all-grade (RR 5.48; 95%CI, 3.76–7.99; *p* < 0.001) and high-grade (5.09; 95% CI: 2.71–9.54, *p* < 0.001) hypertension in comparison with controls [129]. Substantially higher risk of high-grade hypertension compared with sorafenib, sunitinib, vandetanib and pazopanib [129]. Modest risk of developing left ventricular systolic dysfunction [18].

AE, adverse event; ATE, arterial thromboembolic events; AXL, Anexelekto; BRAF, serine/threonine-protein kinase B-raf; c-KIT, tyrosine-protein kinase KIT; c-MET, tyrosine-protein kinase Met; CR, complete response; CRAF, RAF proto-oncogene serine/threonine-protein kinase; CSF-1; colony-stimulating factor 1; DBP, diastolic blood pressure; FLT-3, Fms-related receptor tyrosine kinase 3; HF, heart failure; HR, hazard ratio; INFα, interferon α; LVEF, left ventricular ejection fraction; mo, months; OS, overall survival; PD, progressive disease; PDGF, platelet-derived growth factor; PDGFR, platelet-derived growth factor receptor; PFS, progression-free survival; PR, partial response; RR, risk ratio; SBP, systolic blood pressure; Tie2, endothelial-enriched tunica interna endothelial cell kinase 2.

## Data Availability

Not applicable.
